# Supplementary material for: What factors enhance students’ achievement? A machine learning and interpretable methods approach
Source: PLoS One. 2025 May 16;20(5):e0323345. doi: 10.1371/journal.pone.0323345 (PMC12083833; doi:10.1371/journal.pone.0323345)
Supplement: Attachment — Submitted filename: Response_to_Reviewers.docx.docx [file pone.0323345.s002.docx]

Dear Editor and Reviewers,

Thank you for your valuable comments. Based on your feedback, we have made the necessary modifications to our manuscript. We hope these changes meet the publication standards of this journal.

Best regards,

Authors

**Editor Comments:**

1. Introduction Clarity:

- (1) Clearly define primary AI models in teaching.
AU comments: Thank you for your comments! It is true that the description of this place was not clear enough before, and we added the basic idea of using AI simulation teaching process in Line 62-66. At the same time, we specify the preferred AI simulation model ‘XGBoost‘on line 212.

- (2) Justify using final exam scores as a reflection of learning willingness.

Thank you for pointing out this issue. After reading this literature and reviewing a significant amount of relevant research, I found that there is limited literature on students' learning willingness. In the small number of existing studies, scales and questionnaires are often used to collect data on students' self-efficacy as an indicator to measure their willingness to learn. However, many of the students referenced in this paper have graduated and left school, and some completed their courses a long time ago. As a result, it is currently challenging to obtain accurate self-efficacy data, and there are no practical conditions to supplement this data at present.

On the other hand, through an in-depth review of relevant literature, we found that another dependent variable, student engagement, is more closely related to our research focus: student behaviors and teaching environment & methods. It is more appropriate to use this index to describe the problem we aim to study. Based on existing literature, student engagement and achievement have a strong positive correlation, which is often directly measured by final exam scores. Numerous experts have conducted extensive research in this area, and there is substantial literature supporting this view. Please refer to lines 69–102 in our supplement for further details.

Therefore, we sincerely apologize and request to change the name of the research object Y indicator to "Student engagement/achievement," which better aligns with our research intentions. Additionally, we apply to modify the title to "What Factors Enhance Student Engagement/Achievement? A Machine Learning and Interpretable Methods Approach," and update the original keyword "student learning willingness" to "student engagement/achievement." In this revision, the original Y indicator name "student learning willingness" in this paper has been changed to "student engagement/achievement."

- (3) Include geographical context in literature review to highlight cultural differences.

Thank you for your valuable comments! Regarding the study of student engagement across regions, we have added a section to the article dedicated to this content (lines 133–152). We have consulted and supplemented new literature (lines 134–144 of the paper). At the same time, the authors of relevant literature, such as the relationships between student engagement/achievement and final exam scores, student behavior, and teaching environment & methods, are summarized from various regions and countries (lines 146–148 of the paper). Through a review of the literature, we find that research on how to effectively improve student engagement/achievement has strong cross-regional universality.

2. Methodological Issues:

- (1) Provide a clear research framework, visualizing model roles and inputs/outputs.

AU comments: Once again, thank you for the comments. In response to the third expert's suggestions for chapter adjustments, we have modified the research framework. Additionally, we have provided a detailed explanation of the elements and processes in the research framework figure, which can be found in lines 225–231 of the paper. The output label is depicted as an input source in Fig. 1 because the data from the output label is required to complete the model's training and to evaluate its performance on the test set.
- (2) Explain and cite reasons for categorizing exam scores and using specific predictors.

AU comments: Thank you! To address this issue, we have added a dedicated section (lines 67–132). We have consulted and supplemented relevant literature on student engagement/achievement and final exam scores, engagement/achievement and student behaviors, and engagement/achievement and teaching environment & methods. Based on existing literature and research, we have explained the reasons for selecting these indicators.
- (3) Address limitations like equating exam results with learning motivation and consider adding self-efficacy measures.

AU comments: Thank you for pointing out this issue. After considering this opinion, we have reviewed a significant amount of relevant literature and found that there is limited research on students' learning willingness. In the small number of existing studies, scales and questionnaires are often used to collect data on students' self-efficacy as an indicator of their willingness to learn. However, many of the students referenced in this paper have graduated and left school, and some completed their courses a long time ago. Currently, it is challenging to obtain accurate self-efficacy data, and there are no practical conditions to supplement this data temporarily.

On the other hand, through an in-depth review of relevant literature, we found that another dependent variable, student engagement, is more closely related to our research focus: student behaviors and teaching environment & methods. It is more appropriate to use this index to describe the problem we aim to study. Based on existing literature, student engagement and achievement have a strong positive correlation, which is often directly measured by final exam scores. Many experts have conducted extensive research on this aspect, and there is substantial literature supporting this view. Please refer to lines 69–102 in our supplement in the Introduction. Therefore, we sincerely apologize and change the name of the research object Y indicator to "Student engagement/achievement," which better aligns with our research intentions. In this revision, the original Y indicator name "student learning willingness" in this paper has been changed to "student engagement/achievement."

3. Model Parameters and Evaluation:

- (1) Clarify the need for changing model parameters after cross-validation.

AU comments: Thanks for the suggestion! We reviewed and supplemented additional literature on grid search and cross-validation in the Empirical Results section (lines 362–379). In response to the second expert's opinion that "exploring results with different parameter settings seems impractical and unnecessary," we have removed the section in the Verification that uses different parameters to verify the robustness of the model.

- (2) Justify dataset split ratios and provide sample details.

AU comments: Thanks for the suggestion! We discussed and analyzed the comparison between the dataset partitioning options of 7:3 and 8:2, and we have supplemented the description of the sample training set and the test set. The additional content is on lines 353–362. Additionally, we have provided performance screenshots of the running results for the 7:3 and 8:2 ratios for comparison.

| 7:3 ratio |  |
| --- | --- |
| 8:2 rario |  |

- (3) Structure results to evaluate and compare model performance (e.g., XGBoost vs. SVM, DT, RF, ANN) with detailed validation methods and experimental values.

AU comments: We have used accuracy, precision, recall, and F1-score to evaluate the XGBoost, SVM, DT, RF, and ANN models, as detailed in lines 443–449. The performance parameters obtained from these five model experiments are listed in Table 4, and the results are explained and compared in the text (lines 458–466). Additionally, in response to the first expert's opinion, we have added an explanation of the above four evaluation indicators and included literature support. This supplementary content can be found in lines 450–457 of the paper. Furthermore, we provide performance screenshots of the experimental results for XGBoost, SVM, DT, RF, and ANN for comparison.

| XGBoost |  |
| --- | --- |
| SVM |  |
| DT |  |
| RF |  |
| ANN |  |

**Reviewer #1:**

Introduction:
(1) Researchers continuously explore the utilization of artificial intelligence in
Education -> small letter in ‘education’

AU comments: Thanks for suggestion! Lowercase substitution from "in Education" to "in education" has been completed throughout the text.

(2) Jia, S. -> can you double check the citation requirement of this journal? I think most journal they only need the last name but not the initial of the first name. If initial such as , S. is not needed, please correct it throughout the manuscript.

AU comments: Thank you for your comments! We have corrected the problem throughout the manuscript.

(3) The model was built using a variety of students’ data sets over three academic years. Through the analysis, the passing rate of the final practical examination has been improved, from about 60% to more than 80%............. I don’t understand, through what analysis and what is the important issue for this analysis? Just the observation of 60% to 80%? Or they identified the underlying reasons and therefore they have applied proper intervention to improve it? If just observation of the % change, it will become nothing special here.

AU comments: Thanks for suggestion! We supplemented the description of the research content in Jasna [25], lines 85-87.

(4) Gunawardena, E. [22] used a multiple regression model to predict final exam scores for students in undergraduate business statistics courses. 366 samples, in the winter semester of 2017. Contains three predictors, namely two classroom tests and homework scores. It is proposed that the final exam as a dependent variable is affected by homework and classroom test factors. …… some incomplete sentences inside, please amend

AU comments: Thanks for suggestion! We have modified the incomplete sentence, in lines 88-90 on page 6 of the paper.

(5) There are currently not many articles that focus on the application of interpretable
    machine learning methods in the field of educational teaching. Some research methods rely on fitting student behavior data into the model, while overlooking the comprehensive impact of teaching methods data on the model output………..Please make some citations and give some examples to support what you claimed

AU comments: Thanks for suggestion! We add literature to support the opinion, which is summarized in lines 69-93 of the paper. The example description has been added in lines 156-162 of the paper.

(6) In the introduction section, all the examples and citations did not provide the nationality / location of the previous studies. I think it is also important to tell the readers the country source of those subjects or studies as the cultural differences between Asia, Australia, Western countries can have a tremendous impact on why some are effective and why some are not effective in enhancing students' learning willingness. If not mentioned, it seems we have made an assumption that all these are the same in all countries in the world but it is not the case in reality.

AU comments: Thank you for your valuable comments! For the study of student engagement across regions, we have added a section dedicated to this content (lines 133–152). We have consulted and supplemented new literature (lines 134–144 of the paper). Additionally, we have summarized the relevant literature on the relationships between student engagement/achievement and final exam scores, student behavior, and teaching environment & methods across different regions and countries (lines 146–148 of the paper). Through a review of the literature, we find that research on how to effectively improve student engagement/achievement has strong cross-regional universality.

（7）In table 1, the left hand side is the type of predictors and outcome variable which are the most important key in this study. However, the introduction section only provided some brief and overall background information. Please write something or relevant theory behind to support why “student behavioral data indicators” or its components are potentially important to learning motivation? Similarly why teaching environment and teaching methods are also potentially important to students learning motivation. Please use some proven theories to support if possible. Otherwise, the use of these indicators or predictors will sound intuitive.

AU comments: Thanks for suggestion! We added two chapters, describing "The relationship between students' behavior and engagement/achievement" and "The relationship between Teaching environment & methods and engagement/achievement”. Literature review is added to the content of the chapters as the support of the viewpoint, and the modification is in the 102-132 lines of the paper.

（8）Categorizing students' actual final exam scores into three groups (0, 1, 2) based
on their final exam scores, corresponding to failing, good and excellent grades
respectively and labeled as "low," "medium," and "high," provides a common measure of theoretical course outcomes and authentically reflects student motivation…………. Please provide some citations and explanations on why students exam performance is equivalent to learning motivation? How about some students who are slow learner that they have put tremendous efforts but only got average or below average results?

AU comments: Thank you for pointing out this issue. After considering this opinion, we have reviewed a significant amount of relevant literature and found that there is not much literature and research on students' learning willingness. In the small number of existing studies, scales and questionnaires are often used to collect data on students' self-efficacy as an indicator to measure their willingness to learn. However, many of the students referenced in this paper have graduated and left school, and some completed their courses a long time ago. Currently, it is difficult to obtain accurate self-efficacy data, and there are no practical conditions to supplement this data temporarily.

On the other hand, through an in-depth review of relevant literature, we found that another dependent variable, student engagement, is more closely related to our research focus: student behaviors and teaching environment & methods. It is more appropriate to use this index to describe the problem we aim to study. Based on existing literature, student engagement and achievement have a strong positive correlation, which is often directly measured by final exam scores. Many experts have conducted extensive research on this aspect, and there is a substantial amount of literature supporting this view. Please refer to lines 69–102 in our supplement in the Introduction. Therefore, we sincerely apologize and as we change the name of the research object Y indicator to "Student engagement/achievement," which better aligns with our research intentions. In this revision, the original Y indicator name "student learning willingness" in this paper has been changed to "student engagement/achievement."

（9）This paper uses the real data recorded by micro-assisted teaching platform in the
daily teaching process of the course "Computer Systems" for students majoring in
Software Engineering at Information Engineering School in 2021, 2022, and 2023. The reason for choosing this course as a representative is that this course is a typical theoretical course with very little hands-on experiments, and it focuses more on the explanation and learning of theories. Therefore, the study of the relationship between the data of this course and students' willingness to learn is representative……………If so, it also means that the subject discipline such as theory based like accounting or computer science, practical based such as sports and physical education can be very different? This should also be at least lightly mentioned in the introduction and here you may want to say to minimize unknown confounding variables or noise affecting the prediction accuracy rather than “Therefore, the study of the relationship between the data of this course and students' willingness to learn is representative”

AU comments: Thanks for the suggestion! The reason we chose this course data as an example for our research is that pure theoretical courses, like "Computer System," tend to be more challenging to engage students due to the lack of practical operations such as programming. Unlike other types of courses that can enhance students' sense of achievement and engagement through hands-on experiments, pure theoretical courses are less interactive and therefore more difficult to promote student engagement and academic achievement. By selecting this challenging course as a starting point, we aim to discuss the worst-case scenario. In the future, further research can be conducted on other computer courses with experiments, building on the conclusions of this case. These reasons have been added and described in lines 194–199 of the paper.

(10) I think there is one major limitation of this study that you can only make an assumption exam result = students’ learning motivation but it may be partially true. You should have another variable called “self-efficacy of learning motivation” so that students’ self-rate their learning motivation. If you can point out the very high correlation between the self-rate / self-efficacy one with the exam result, it will be more persuasive to use the exam result high medium and low to indicate high, middle and low motivation of students. Please highlight this in the discussion as your limitation.

AU comments: Thanks for the suggestion! After considering this opinion, we have reviewed a significant amount of relevant literature and found that there is not much research on students' learning willingness. In the small number of existing studies, scales and questionnaires are often used to collect data on students' self-efficacy as an indicator to measure their willingness to learn. However, many of the students referenced in this paper have graduated and left school, and some completed their courses a long time ago. Currently, it is difficult to obtain accurate self-efficacy data, and there are no practical conditions to supplement this data temporarily.

On the other hand, through an in-depth review of relevant literature, we found that another dependent variable, student engagement, is more closely related to our research focus: student behaviors and teaching environment & methods. It is more appropriate to use this index to describe the problem we want to study. Based on existing literature, student engagement and achievement have a strong positive correlation, which is often directly measured by final exam scores. Many experts have conducted extensive research on this aspect, and there is substantial literature to support this view. Please refer to the content on lines 69–102 in our supplement in the Introduction. Therefore, we sincerely apologize and change the name of the research object Y indicator to "Student engagement/achievement," which is more in line with our research intention. In this revision, the original Y indicator name "student learning willingness" in this paper has been changed to "student engagement/achievement."Top of FormBottom of Form

(11) The section of XGBoost Model is good in explaining the model logic and formula things from a technical point of view. However, if this paper is more on the education side rather than computer science side, I suggest on top of these explanation, you can add few example from previous literature how XGBoost classifier successful do prediction in education or relevant areas so that readers can understand it may be good fit to certain kind of data structure / nature.

AU comments: Thanks for suggestion! We add some literature reviews on XGBoost classifier successful do prediction in education at lines 266-278.

(12) SHAP section – please add at least one more citation to support its usefulness。

AU comments: Thanks for suggestion! We have supplemented the literature support in lines 332-338 of the paper.

(13) Please tell the readers if any data pre-processing, feature selection procedures (e.g. RFE or MRMR) used and any hyper-parameter tuning (e.g. gridsearch or random search?).

AU comments: Thanks for suggestion! We have added the description of data pre-processing in lines 347-350 of the paper. Grid search and cross-validation methods are used to deal with the optimization of model parameters (lines 361-362), and the literature in this area is further supplemented (lines 362-379).

(14) Please give either a very brief explanation on Ai metrics such as F1 score, confusion matrix…etc. with at least 1 citation

AU comments: Thanks for suggestion! We add explanation of the above four evaluation indicators and add literature support. The supplementary content is in lines 448-455 of the paper.

(15) Why 7:3 split but not common one 8:2 ? (and how many training and how many testing set in total?)

AU comments: Thanks for the suggestion! We discussed and analyzed the comparison between the dataset partitioning options of 7:3 and 8:2, and we have supplemented the description of the sample training set and test set. The supplementary content can be found on lines 353–362. Additionally, we provide performance screenshots of the running results for the 7:3 and 8:2 ratios for comparison here.

| 7:3 ratio |  |
| --- | --- |
| 8:2 rario |  |

(16) The section below figure 4 explaining the SHAP is very detailed and technical. It can be more precise instead of an article teaching people the interpretation of SHAP values in detail.

AU comments: Thanks for the suggestion! Due to the reordering of the revised chapters, the original Figure 4 has been updated to Figure 6. We have added further interpretation of the SHAP value results presented in Figure 6 in lines 598–610 of the paper.

(17) The whole discussion only focused on repeating the description of the results such as which one is more important, and which one is less. However, it lacks an in-depth discussion on WHY homework assignments, WHY discussion activities, and why game-based or storytelling things from the education point of view. As the educational-related manuscript or paper rather than a computer science or visualization paper, the discussion section should give more insight and be based on previous educational literature to make comparisons and propose explanations as well as provide future study direction. Now it sounds like a paper teaching the interpretation of data visualization, especially SHAP rather than a true education related paper.

AU comments: Thanks for suggestion! In the discussion part, combined with the results of model simulation and analysis, we further added consideration in teaching, which is provided in lines 659-676 of the paper.

**Reviewer #2:**

Thank you for your encouragement! From your suggestions, we have a lot of good ideas, thank you very much!

（1） It is not clear in the introduction which are the primary models for the integration of AI in teaching.
    “A comprehensive analysis of educational artificial intelligence, as outlined in literature, has identified three primary models for the integration of artificial intelligence in teaching…”

AU comments: Thank you for your comments! You are correct that the previous description was not clear enough. We have added a basic model for using AI to simulate the teaching process in lines 62–66.

(2) Consider using 'opaque box' instead of 'black box' to describe the model, as it avoids potential negative connotations and supports more inclusive language

AU comments: Thanks for suggestion! Substitution from "black box" to "opaque box" has been completed throughout the text.

(3) The use of "On the other hand" seems inappropriate as it introduces a new idea that does not contrast with the previous one.
    “On the other hand, to achieve the ideal learning outcomes, teachers have tried various teaching methods to promote students' willingness to learn, such as using game-based methods…"”

AU comments: Thanks for suggestion! We have removed the inappropriate "On the other hand" in line 128.

(4) The indicators should not be considered part of the main contributions as they were not formalized (maybe beacuse may not be necessary). They could be discussed as a marginal contribution in the discussion section. It is also important to clarify the period used to create frequency variables. Weeks? Months? Years?

AU comments: Thanks for the suggestion! We have revised the description of specific indicators in the contribution section of the Introduction (the modification is in lines 208–210 of the paper) and discussed it in the Discussion section. We have also added the period for frequency variables in Table 1 and other relevant parts of the paper, specifying that it is "in a semester."

(5) This statement requires a reference.
    “Because final exam score are the most intuitive reflection of a student's willingness to learn a course...”

AU comments: Thank you for pointing out this issue. After considering this opinion, we reviewed a significant amount of relevant literature and found that there is not much research on students' learning willingness. In the small number of existing studies, scales and questionnaires are often used to collect data on students' self-efficacy as an indicator to measure their willingness to learn. However, many of the students referenced in this paper have graduated and left school, and some completed their courses a long time ago. Currently, it is difficult to obtain accurate self-efficacy data, and there are no practical conditions to supplement this data temporarily.

On the other hand, through an in-depth review of relevant literature, we found that another dependent variable, student engagement, is more closely related to our research focus: student behaviors and teaching environment & methods. It is more appropriate to use this index to describe the problem we want to study. Based on existing literature, student engagement and achievement have a strong positive correlation, which is often directly measured by final exam scores. Many experts have conducted extensive research on this aspect, and there is substantial literature to support this view. Please refer to the content on lines 69–102 in our supplement in the Introduction. Therefore, we sincerely apologize and change the name of the research object Y indicator to "Student engagement/achievement," which is more in line with our research intention. In this revision, the original Y indicator name "student learning willingness" in this paper has been changed to "student engagement/achievement."

(6) The course chosen for analysis may not be representative due to its theoretical difficulty and low pass rate. Suggest changing the text to present it as a good option to be a starting point, with future work needed to use other courses for comparison and better external validation

AU comments: Thank you for helping us broaden our thinking! The reason we chose this course data as an example for our research is that teaching a pure theoretical course tends to be more monotonous due to the lack of practical operations, such as programming, compared to other courses. Other types of courses can enhance students' sense of achievement and engagement more effectively through hands-on experiments, like building hardware or software. As a result, promoting student engagement and academic achievement in a pure theoretical course is more challenging. Therefore, we chose the most challenging scenario as a starting point for discussion, using the pure theoretical course "Computer System" as an example. In the future, further research can be conducted in the teaching of other computer courses with experiments, building on the conclusions of this case. The reasons have been added and described in lines 194–199 of the paper.

(7) What problems? Are they related to the limited interpretability as XGBoost feature importance only provides scores rather than detailed explanations?
    “To address the problem of interpretability associated with feature importance in XGBoost,...”

AU comments: Thanks for suggestion! The wording here is indeed inappropriate and has been deleted from the text.

(8) Local explanations may be unnecessary for achieving the paper's goals as outlined in the first chapter. The section related to the local explanations could be omitted since the paper initially seeks global explanations.

AU comments: Thanks for the suggestion! Local explanations support decision-makers by detailing the impact of each feature on the model's decision-making process. When explaining a solitary prediction, SHAP provides robust data visualization capabilities for presenting the explanation results. However, the relationship between each factor and the predicted result remains unclear within the opaque box of interpretation model prediction, which is a key focus of this paper. Therefore, we have retained this section and included it in the empirical results to provide a more intuitive presentation and explanation of how each factor affects the forecast results. At the same time, we simplified the wording. The revised content is in lines 480–523.

(9) Why did the authors arbitrarily change the model parameters that had been adjusted in advance through a cross-validation process? What is the practicality of exploring alternative parameter settings to compare model output? In other words, since the model parameters were properly adjusted earlier, exploring results with different parameter settings seems impractical and unnecessary. If the intent is to demonstrate model robustness and generalizability, simply changing parameters arbitrarily seems insufficient. A systematic approach would be more appropriate. I recommend omitting this part or explaining better its intention and need.
    “The XGBoost model mentioned earlier achieved an accuracy of 85.19%. To validate the effect of adjusting the primary parameter range on accuracy, the optimal parameters were adjusted accordingly, such as the learning rate was decreased from 0.3 to 0.1...”

AU comments: Thanks for the suggestion! Previously, we adjusted the main parameters as a way to verify the robustness of the model, a practice that has been referenced in other literature. After considering this opinion, we consulted and supplemented more literature on grid search and cross-validation (lines 362–379 of the paper). According to the principles of the grid search method, the result is already the optimal parameter. After further reading, we also agree that adjusting parameters to verify the robustness of the model is not suitable. Therefore, we have removed the use of different parameters to verify the robustness of the model from the validation section.

(10) Replace Figure 7 with a table.

AU comments: Original Figure 7 was part of the section on adjusting the main parameters to verify the performance of the model. Since this content has been removed, Figure 7 has also been deleted.

(11) Comparing feature importance rankings from XGBoost directly with SHAP explanations is promising, as they are computed differently. Within this context, similarities could provide more evidence about the true role of variables in the data-generating process. This aspect could be more detailed and more explored in the text.

AU comments: Thank you for your advice and help us point the way! We have detailed the description of this part of the content, added the practical significance of this result, and revised the content in lines 538-550 of this paper.

(12) Use formal tables instead of figures to present model outputs such as in Fig. 7 and 9.

AU comments: Original Figure 7 belonged to the section on adjusting the main parameters to verify the performance of the model. With that content removed, Figure 7 has also been deleted. Additionally, original Figure 9 has been converted to a table and renamed as Table 5 (line 561 of the paper).

(13) In the conclusion, highlighting the six most important variables is excessive. The models seem to use only four variables for predictions, as indicated by the first Global SHAP explanations and feature importance rankings in the first model. The other variables have marginal explanation power and their ranking could be only due to chance.

AU comments: Thanks for the suggestion! The description of the six indicators has been streamlined and revised in the conclusion.

(14) The variables have different names throughout the text and in the figures. Please standardize them.

AU comments:

Thanks for the suggestion! We have searched and reviewed the names of the 10 input factors and 1 output label throughout the entire paper and have made the necessary modifications.

**Reviewer #3:**

1) Overall structure of the manuscript:
   - Please consider restructuring the article according to the content sections like the
   normal structure of an article. For example, section 3. (“Comparison and Evaluation”) and section 4. (“Explanation and Testing”) seem to belong to the Results section.
   AU comments: Thank you for your advice！We have adjusted the structure of the paper and classified the contents presented in the usual results section into a chapter named "3 empirical Results"; Categorize Verification in the "4 Verification" section.

- Section 2. (“Research framework, Datasets and Methods”) should be renamed to “Methodology” to encompass all the relevant content.

AU comments: Thank you for suggestion！We have renamed the original chapter 2, "Research framework, Datasets and Methods," to "Methodology."
- Please clearly define research questions. Subsequently, align the Methodology, Results, and Discussion sections to address these research questions.

AU comments: Thank you for your advice！We summarized and supplemented the content of Research objective in lines 168-175 of the paper. In addition, part of the Methodology, Results, and Discussion sections are modified and supplemented, and revised in lines 218-229, 639-685, and 691-711 of the paper.

2) Formating of manuscript:
   - Please ensure consistency in the formatting of titles, images, and tables throughout
   the manuscript.

AU comments: Thank you for suggestion！We have corrected this issue.
- Some images such as Fig. 7 and Fig. 9 need to be considered for more appropriate
expression.

AU comments: Thank you for the suggestion! In line with the second expert's opinion, the original Figure 7, which belonged to the section on adjusting the main parameters to verify the performance of the model, has been deleted along with that section. Additionally, the original Figure 9 has been converted into a table and renamed as Table 5 (line 561 of the article).

3) Research frameworks:
   - Please clarify the rationale behind considering the "final exam score as the most intuitive reflection of students' willingness to learn a course."

AU comments: Thank you for pointing out this issue. After considering this opinion, we reviewed a significant amount of relevant literature and found that there is not much research on students' learning willingness. In the small number of existing studies, scales and questionnaires are often used to collect data on students' self-efficacy as an indicator to measure their willingness to learn. However, many of the students referenced in this paper have graduated and left school, and some completed their courses a long time ago. Currently, it is difficult to obtain accurate self-efficacy data, and there are no practical conditions to supplement this data temporarily.

On the other hand, through an in-depth review of relevant literature, we found that another dependent variable, student engagement, is more closely related to our research focus: student behaviors and teaching environment & methods. It is more appropriate to use this index to describe the problem we want to study. Based on existing literature, student engagement and achievement have a strong positive correlation, which is often directly measured by final exam scores. Many experts have conducted extensive research on this aspect, and there is substantial literature to support this view. Please refer to the content on lines 69–102 in our supplement in the Introduction. Therefore, we sincerely apologize and change the name of the research object Y indicator to "Student engagement/achievement," which is more in line with our research intention. In this revision, the original Y indicator name "student learning willingness" in this paper has been changed to "student engagement/achievement."

- Please provide a clear basis for the research framework. Consider visualizing the role of each model in the procedure, specifying the input and output for each model. Fig. 1 appears confusing as it shows all factors and output labels as input sources of the models; a more detailed description of each element within the framework is needed.

AU comments: Thank you for suggestion！Combined with the above adjustment of the overall structure and chapters of the article, we modified the research framework in Figure 1. At the same time, the elements and processes in the research framework figure 1 are explained in words, which are 223-229 lines of the paper. The output label is drawn as input source of the model in Fig.1 because the data of output label is required to complete the training of the model and evaluate the performance of the model testing on test set.

4) Datasets, considered to provide details about the number of samples used for training and testing in this section (currently mentioned only in the results interpretation).

AU comments: Thank you for suggestion！In this section, we have added detailed information on the number of samples used for training and testing in lines 249-250 of the paper.

5) Methods: As stated above, please consider adjusting the structure of this section to clearly outline each method used to answer each research question.

AU comments: Thank you for the suggestion! The two main methods used in this paper are the XGBoost model and the SHAP interpretation method, which are consistent with the previous descriptions. Therefore, the structure of the methods chapter has not been adjusted in this revision, but the expression of SHAP has been improved and modified (in lines 319–328).

6) Results:
   - The structure of section 3. (“Comparison and Evaluation”) might be confusing. It
   would be better to first evaluate the performance of the XGBoost model and then compare it with other models (SVM, DT, RF, and ANN). Also, detail the validation method used to evaluate the models' performance.

AU comments: Thank you for the suggestion! We have renamed Chapter 3 to "Empirical Results" and adjusted the order of the content. The revised chapter first describes the modeling steps, then evaluates the performance of the XGBoost model, followed by a comparison with other models (SVM, DT, RF, and ANN). Finally, the chapter concludes with an "inside" analysis of the results. These changes have been made in lines 339–464.

- For Tabs. 3, please provide some experimental values to ensure the values are optimal, potentially through ablation studies.

AU comments: Thank you for the suggestion! After considering this opinion, we consulted literature on grid search and cross-validation. According to the principles of the grid search method, the calculated parameter result is already the optimal parameter. Therefore, we added the relevant literature as theoretical support in the paper (lines 362–379) to explain the optimization problem of parameters. Additionally, the code we uploaded includes the grid search and cross-validation process.
- For Fig. 2, based on the matrix, please explain why the error rate is higher for high true labels compared to lower labels.

AU comments:

Thank you for the suggestion! We have supplemented the analysis and literature regarding the reasons why the error rate is higher for high true labels compared to lower labels (lines 417–424).
- Please provide a more detailed analysis of the results shown in Fig. 7 to 9 to support your claims.

AU comments:

With the opinion of the second expert, the original Figure 7, which belonged to the section on adjusting the main parameters to verify the performance of the model, has been deleted along with that section. The original Figure 8, "shap_value_importance figure," has been modified and is now Figure 5 (line 537 of the paper). In line with the second expert's suggestions, the original Figure 9, "Feature importance ranking of disrupting single column data," has been converted to Table 5 (line 561 of the paper). We have added a more detailed analysis of the revised Figure 5 and Table 5. These changes are reflected in lines 541–550, 552–560, and 562–571.
