# Supplementary material for: What factors enhance students’ achievement? A machine learning and interpretable methods approach
Source: PLoS One. 2025 May 16;20(5):e0323345. doi: 10.1371/journal.pone.0323345 (PMC12083833; doi:10.1371/journal.pone.0323345)
Supplement: Attachment — Submitted filename: Response to Reviewers 12.5e.docx [file pone.0323345.s003.docx]

Dear Editor and Reviewers,

Thank you for your valuable feedback. We have carefully reviewed your comments and made the necessary revisions to our manuscript accordingly. We hope that the changes meet the publication standards of the journal.

Please note that the modifications made in response to the reviewers' comments are highlighted in yellow in the marked version. Additionally, any language edits have been tracked using the track changes feature.

We would also like to acknowledge the assistance of Associate Professor Thomas Stephen Ramsey, a native speaker, who kindly helped us with the language editing of the manuscript.

Thank you once again for your time and consideration.

Best regards,

Authors

**Editor Comments:**

- 1. The link between student engagement and achievement needs further clarification.
AU comments: Thank you for pointing out this issue. We appreciate your feedback, and we have made several refinements to clarify the connections between engagement, achievement, and the relevant variables. Firstly, we have refined the data and supporting evidence from the literature to better illustrate the positive correlation between engagement and achievement (lines 62-102). In reviewing the literature, we found that most studies use the Pearson correlation coefficient (r) to describe this relationship, while others employ the Goodness of Fit Index (GFI = 0.980) or Cohen's d. This updated presentation provides a clearer understanding of the relationship between engagement and achievement.

Secondly, we have further clarified the dependent variables in the study. Specifically, we now express Y as "achievement," which we believe more accurately reflects the outcome of interest. Consequently, we have removed "/engagement" from the original title (line 1), ensuring that the focus is on achievement as the primary dependent variable.

Finally, we have reorganized the relationships between student behavior, engagement, achievement, and final exam scores. In line with previous literature, we acknowledge that student achievement is often measured by final exam scores (line 105), while student behavior is used as an indicator of engagement (line 106). Additionally, to broaden the scope of our analysis, we have included indicators related to the teaching environment and methods as independent variables. We discuss their impact on student achievement in lines 143-147, providing a more comprehensive view of the factors that influence achievement.

- 2. improvements to the manuscript’s structure and writing style are required to enhance its overall clarity and accessibility.

AU comments: Thank you for pointing out this issue. We have made several adjustments to improve the clarity and accessibility of the manuscript. Firstly, we restructured the content to enhance its flow. Specifically, the section on indicator selection has been moved to the second chapter, now titled “Indicator Selection” (line 238), to create a more logical organization of the material.

Secondly, in response to Reviewer #4’s comment that “some machine learning concepts are not explained in enough detail for readers who may be unfamiliar with the field, limiting the paper’s accessibility to a broader audience,” we have added an explanation of machine learning modeling in lines 209-213. This addition aims to provide clearer context and make the paper more accessible to readers with varying levels of familiarity with machine learning.

Lastly, in response to Reviewer #1’s suggestion to enhance the writing of the manuscript, we carefully reexamined the structure and wording throughout the entire paper. We also asked native speaker professor Thomas Stephen Ramsay of School of Foreign studies, China Three Gorges University to review our writing and he made extensive correction on the manuscript to refine the language and improve the overall quality of the manuscript.

**Reviewer #1:**

1. Line 26 to 27, line 194 to 199 and line 237 to 242  - To say pure theory course as most boring can be dogmatic and it does not sound nice.

AU comments: Thanks for your suggestion! We removed the original description on previous line 26 to 27 and previous line 237 to 242, and refined description on line 188 to 189 as you suggested. Thanks again!

2. Line 88 - may delete "2017", sounds a bit weird.

AU comments: Thank you for your comments! We have deleted “2017” on line 86.

3. You have used many citations to support the strong association between students' engagement and academic performance/achievement, can you highlight a bit the findings (e.g. correlation ?) from those studies? You may use for example (r=0.90) or R2 values if it was regression, to supplement the findings from previous studies when you highlighted those variables. I think this is quite important as you have made an assumption exam results ~ engagement. You need to show the very high validity between these or otherwise the topic and study findings can no longer be valid as you can't use exam result to reflect students engagement anymore.

AU comments: Thank you for your insightful suggestion. We agree that the description in this section was not sufficiently clear previously. To address this:

We have refined the data and evidence in the literature to better illustrate the positive correlation between engagement and achievement (lines 62-102). Specifically, we highlight that most studies use the Pearson correlation coefficient (r) to describe this relationship, while one study employs the goodness of fit index (GFI = 0.980), and another utilizes Cohen's d. We have further clarified the dependent variable (Y). Upon reflection, we believe that "achievement" is a more accurate term to describe Y, so we have updated the original title (line 1) by removing "/engagement."

We have restructured the explanation of the relationships between student behavior, engagement, achievement, and final exam scores. In line with prior literature, student achievement is often measured directly through final exam scores (line 105), and student behavior is commonly used as an indicator of engagement (line 106). Therefore, in addition to engagement data (such as student behavior), we have incorporated indicators of teaching environment and methods as additional independent variables, exploring their impact on student achievement (lines 143-147).

4. Line 184 - means --> reflects

AU comments: Thanks for your suggestion! We have modified “means” to “reflects” on line 247.

5. Line 222 to 229 - I think you should use past tense throughout?

AU comments: Thanks for your suggestion! We modified description with past tense throughout line 218 to 222.

6. Line 249 - 314 (70%)..........135 (30%).

AU comments: Thanks for your suggestion! We refined the description on line 236-237 and line 367.

7. Line 472 - represent

AU comments: Thanks for your suggestion! We refined the description on line 476.

8. However, the writing of this manuscript can be further enhanced (e.g. by a native English professional writer). Although most or nearly all sentences are readable, the paragraphs arrangement, sentence structure and writing style are somewhat clumsy, with sentences redundant or duplicated sometimes. I did never see a conclusion with more than 1 page in other manuscripts (if so, it is either not precise and well written enough or some contents should be in discussion rather than conclusion). Meanwhile some contents or similar sentences existed in many places repeatedly in introduction, method/results and discussion/conclusion which are not necessary.... as it finally made your manuscript necessarily long (732 lines now). Therefore, it is recommended to further enhance the writing style before finally getting accepted.

AU comments: Thanks for your suggestion! (1) We carefully rechecked and refined the structure and wording throughout the entire manuscript to ensure clarity and coherence. (2) We revised the conclusion by eliminating repetitive descriptions and making the key points more concise. Additionally, we also asked native speaker professor Thomas Stephen Ramsay of School of Foreign studies, China Three Gorges University to review our writing and he made extensive correction on the manuscript to refine the language and improve the overall quality of the manuscript. Track change marks the English language being edited.

**Reviewer #4:**

The paper presents several challenges in its current form, which detract from its clarity. There are instances where the argumentation appears confused, making it difficult for readers to follow the line of reasoning. The link between achievement and engagement is insufficiently developed and explained, weakening the argument's depth and making it hard to understand how these two variables relate in the context of the study.
AU comments: Thank you for your valuable comments! We acknowledge that the description of this section was not clear enough before. To address this:

We have reorganized the relationships between student behavior, engagement, achievement, and final exam scores. As noted in previous literature, student achievement is often directly measured by final exam scores (line 105), while student behavior typically serves as a measure of engagement (line 106). To improve clarity, we have included additional indicators related to teaching environment and methods as part of the independent variables, and we discussed their impact on student achievement (lines 143-147).

We further clarified the dependent variables (Y), which we now believe are more accurately represented as “achievement.” As a result, we have removed “/engagement” from the original title (line 1) to better reflect the focus on student achievement.

We have refined the data and evidence in the literature to better illustrate the positive correlation between engagement and achievement (lines 62-102). Most of the studies cited in the literature use the Pearson correlation coefficient (R) to describe this relationship. Additionally, one study uses the goodness of fit index (GFI = 0.980), and another uses Cohen’s d as a measure of effect size.

2. Furthermore, some machine learning concepts are not explained in enough detail for readers who may be unfamiliar with the field, limiting the paper’s accessibility to a broader audience.

AU comments: Thanks for your suggestion! We add explanation of Machine learning modeling on line 209-213 to enhance accessibility to a broader audience.

3. Additionally, an ordinal response variable is treated as a categorical one, which could lead to misinterpretation of the data and undermines the validity of the results.

AU comments: Thank you for pointing out this issue! We added a quantitative difference description of the classification to the categorical variable definition (on line 252-256).
