# Supplementary material for: What factors enhance students’ achievement? A machine learning and interpretable methods approach
Source: PLoS One. 2025 May 16;20(5):e0323345. doi: 10.1371/journal.pone.0323345 (PMC12083833; doi:10.1371/journal.pone.0323345)
Supplement: Attachment — Submitted filename: Response to Reviewers 2.3.docx [file pone.0323345.s004.docx]

Dear Editor and Reviewers,

Thank you for your valuable comments. Based on your feedback, we have made the necessary modifications to our manuscript. We hope these changes meet the publication standards of this journal.

Best regards,

Authors

**Editor Comments:**

1.- Abstract
• The opening sentence is unclear and needs to be rewritten for better readability and impact.
AU comments: Thank you for pointing out this issue. We have refined descriptions on line 16 to 18 as you suggested.

2. • From line 22, starting with “The results show…,” restructure this section to present the methodology first, followed by the results. The current arrangement is confusing, as it alternates between these elements.

AU comments: Thank you for pointing out this issue. We have adjusted on line 23-25 to improve the clarity and accessibility of the manuscript. Thanks a lot!

3. Literature Review
   • Specify the type of courses being analyzed (e.g., in-person, online, blended) and the educational level (e.g., K-12, university).

AU comments: Thank you for pointing out this issue. We have checked each cited literature and added corresponding descriptions of the course types and educational levels. The modifications are located at lines 62-63, 66-68, 72, 76-77, 79-80, 84-85, 97, 100, 102, 106, 117-118, 120-121, 125-126.

4. Chapter 1.2.1
   • Following revisions, the focus of the study now appears to be on "student achievements." Revise this section to better align with the updated focus. Is it necessary all the discussion?

AU comments: Thank you for insightful suggestion! We do feel that this part is overelaborated and lacks strong pertinence. Meanwhile, taking into account the comments of Reviewer #1, We removed the original description on previous line 76 to 85 and previous line 97 to 102, and refined description on line 72 to 75. Thanks a lot!

5. Chapter 1.4
   • Expand the explanation of the “opaque box” and provide supporting citations.

AU comments: Thank you for pointing out this issue. We expanded the so-called "opaque box" with more comprehensible expressions and added reference to literature regarding this point on line 157-158.

6. Chapter 1.5
   • Clearly specify the characteristics of the course: Is it in-person or online? What is the educational level? How are examinations conducted?

AU comments: Thank you for pointing out this issue. We have added descriptions of the course type, students' educational level, and the forms of final exams on line 176-178.

7. Methodology
    (1) Add citations when introducing machine learning methods.

AU comments: Thank you for pointing out this issue. We have added citations when introducing machine learning methods on line 197.

8. (2) Justify the division of the dataset into 7:3 or 8:2 splits with appropriate references.

AU comments: Thank you for pointing out this issue. We have searched for more literature on artificial intelligence applications and found that both the 8:2 and 7:3 ratios are common choices in current literature. The ratio can be adjusted according to the actual data to balance the practical needs of model training and evaluation. The modifications are located at lines 409-411.

9. (3) Specify the grading scale of the test (e.g., 0 to 100).

AU comments: Thank you for pointing out this issue. We have further supplemented the description of the question - setting, scope, and scoring standards of the final exam, as well as the criteria that teachers customarily use to grade students' final - exam scores, on line 236-243.

10. (4) Provide detailed explanations of the indices used for model comparison (e.g., accuracy, F1 score).

AU comments: Thank you for pointing out this issue. We have added a section "2.4.4 Metrics for comparing model performance" to explain Accuracy/Precision/Recall/F1_score, at lines 354-366.

11. (5) On line 402, when discussing “Traditional feature importance…,” include citations to strengthen this point.

AU comments: Thank you for pointing out this issue. We have added citation of relevant literature at the part "Traditional feature importance…" in the text, which is at line 338.

12. Discussion
    • Relate the results more explicitly to existing literature to better contextualize the findings and strengthen the paper’s contribution.
    AU comments: Thank you for pointing out this issue. We have related the results to relevant existing literature on line 664-665, 672, 674-676, 677-679, 681-682, 687.
13. particularly methodological concerns regarding the treatment of the ordinal response variable.

AU comments: Thank you for your valuable comments! Although current literature shows that in various fields, different machine learning models, rather than specialized models for ordinal response variables, have been used to simulate and predict the classification of ordinal response variables ([44]-[46], line 300-306), we note that our output labels (0, 1, 2), which correspond to failing, passing, and achieving good grades, represent an ordinal response variable. Therefore, we believe it's necessary to further investigate models specifically designed for ordinal data, like the Ordinal Forest.

So, we read literature Reviewer#4 provided and some other literature on the handling of ordinal response variables. Then we added a section titled "2.4.2 Handling of ordered response variables", which introduces the methods and models for handling ordinal response variables in the current literature (in lines 299-322 of the text). In addition, we used the specialized model Reviewer#4 recommended, namely the Ordinal Forest model in R language [52], for simulation and prediction, and compared the simulation accuracy with that of other models (lines 373-375 and 378-391).

Exhibited in Table 2 are the assessments of the test set's predictive performance across various models (at line 379-383).

**Table 2 Comparison of the prediction results**

| Model Name | Accuracy | Precision | Recall | F1-score |
| --- | --- | --- | --- | --- |
| SVM  DT  ANN  OF(Ordinal Forest)  XGBoost(ignore ordered response variable)  **XGBoost(add ordered response variable)** | 0.725926  0.777778  0.829630  0.888889  0.888889  0.922222 | 0.798498  0.771750  0.851846  0.896552  0.884175  0.936111 | 0.695617  0.764443  0.821073  0.838710  0.879529  0.907571 | 0.718926  0.767545  0.832432  0.866667  0.881567  0.919111 |

Below figure is a screenshot of the operation and result of the OF model in R language, and the code has been uploaded to GitHub.doi:<https://doi.org/10.5281/zenodo.14788957>.

Next is a screenshot of the operation results of the XGBoost model that didn't specifically handle ordinal response variables before.

From Table 2, we found that the prediction accuracy of Ordinal Forest model in R language [52] is better than that of the SVM, DT, and ANN models which do not consider the handling of ordinal response variable. However, its accuracy is equal to that of the XGBoost model which does not consider the handling of ordinal response variable.

So we added a function for handling ordinal response variable [49, 50] to the XGBoost model, the code has been uploaded to GitHub. doi:<https://doi.org/10.5281/zenodo.14788957>.

This time, we found the accuracy of the XGBoost model (add the handling of ordered response variable) was significantly improved. The following figure is a screenshot of the operation results of the XGBoost model with the handling of ordinal response variables added.

Since the XGBoost model (add the handling of ordered response variable) has demonstrated better prediction performance than other models, we still recommend the XGBoost model. All the subsequent figures in the text have been regenerated and modified according to the new XGBoost model.

Your suggestion has significantly improved the accuracy of our simulation and prediction. Thanks a lot!

**Reviewer #1:**

1. Line 76 - 107: When using r value, the correlation coefficient to support e.g. the academic achievement is associated with the engagement, we need to pay attention to the actual r value. Even though it is statistically significant such as p < 0.05, but if the r value is just 0.1-0.4, we should not say it is strong association, correlation or connection. For those value below 0.3, I may not present them in the introduction. Even 0.3-0.5 only indicates moderate correlation/association but still better than 0.1-0.29. As you want to tell the readers that they have some relationship or good association but the findings from previous studies showing r = 0.17, 0.28, 0.14, 0.24....etc. These on the other hand are somewhat contradicting to what you said. We may need to tell the truth in a strategic and smart way. E.g. Previous studies showed the association between the academic performance and student engagement through the student behavior (i.e. "Distinct days active" and "Total number of posts made in discussion forums") of MOOCs platform (r=0.32 to 0.38) [21]. Similarly, please review line 102-106, I will only choose the amount of work done r = 0.30 (2 significant difference) but not to show others with r value <0.3 as the correlations were so weak to support your statement/arguments.

AU comments: Thanks for your insightful suggestion! Your suggestion articulates precisely what we intended to convey. What we intended to express previously was that there is a clear connection between student achievement and student engagement. This connection is manifested through the relationships between achievement and student behaviors such as “submission rate,” “on - time rate,” “extra attempts,” and “self - reported time,” among others. However, there were indeed the issues you raised in our previous expression. Meanwhile, considering the comments of the academic editor: “Chapter 1.2.1：Following revisions, the focus of the study now appears to be on "student achievements." Revise this section to better align with the updated focus. Is it necessary all the discussion?” We removed the original description from previous line 76 to 85 and previous line 97 to 102, and refined description on line 72 to 75 as you suggested. Thanks a lot!

2. Line 85 - 90: The effect size using Cohen's d is to assess the magnitude of difference between two groups. It is NOT an assessment of the correlation. Therefore, it is a flaw or misunderstanding to say using Cohen's d to show a positive linear relationship. Moreover, I have checked the paper by Phebe [23] that it does not have the d value 2.05 very large effect. Please check carefully and also clarify this part.

AU comments: Thank you for your insightful comments! We indeed had a misunderstanding of Cohen's d previously. Thank you for pointing it out! Meanwhile, considering the comments of the academic editor: “Chapter 1.2.1：Following revisions, the focus of the study now appears to be on "student achievements." Revise this section to better align with the updated focus. Is it necessary all the discussion?” We removed the original description from previous line 80 to 84. Thanks a lot!

**Reviewer #4:**

The primary issue with this manuscript lies in the treatment of the ordinal response variable as categorical. This is a critical methodological flaw that undermines the reliability and interpretability of the results. To address this: I strongly recommend exploring models specifically designed for ordinal data, such as ordinal Forest (refer to: <https://link.springer.com/article/10.1007/s00357-018-9302-x)>.

Include comparison indices suitable for ordinal responses to ensure the analysis is rigorous and appropriate for the data type.
examples:
- Ballante, E., Figini, S., and Uberti, P. (2022). A new approach in model selection for ordinal target variables. Computational Statistics, 37(1):43–56.
- Cardoso, J. S. and Sousa, R. (2011). Measuring the performance of ordinal classification. International Journal of Pattern Recognition and Artificial Intelligence,25(08):1173–1195.
In my opinion, until this fundamental concern is addressed, the validity of the findings remains questionable.

AU comments: Thank you for your valuable comments! Although current literature shows that in various fields, different machine learning models, rather than specialized models for ordinal response variables, have been used to simulate and predict the classification of ordinal response variables ([44]-[46], line 300-306), we note that our output labels (0, 1, 2), which correspond to failing, passing, and achieving good grades, represent an ordinal response variable. Therefore, we believe it's necessary to further investigate models specifically designed for ordinal data, like the Ordinal Forest.

So, we read the literature you provided and some other literature on the handling of ordinal response variables. Then we added a section titled "2.4.2 Handling of ordered response variables", which introduces the methods and models for handling ordinal response variables in the current literature (in lines 299-322 of the text). In addition, we used the specialized model you recommended, namely the Ordinal Forest model in R language [52], for simulation and prediction, and compared the simulation accuracy with that of other models (lines 373-375 and 378-391).

Exhibited in Table 2 are the assessments of the test set's predictive performance across various models (at line 379-383).

**Table 2 Comparison of the prediction results**

| Model Name | Accuracy | Precision | Recall | F1-score |
| --- | --- | --- | --- | --- |
| SVM  DT  ANN  OF(Ordinal Forest)  XGBoost(ignore ordered response variable)  **XGBoost(add ordered response variable)** | 0.725926  0.777778  0.829630  0.888889  0.888889  0.922222 | 0.798498  0.771750  0.851846  0.896552  0.884175  0.936111 | 0.695617  0.764443  0.821073  0.838710  0.879529  0.907571 | 0.718926  0.767545  0.832432  0.866667  0.881567  0.919111 |

Below figure is a screenshot of the operation and result of the OF model in R language, and the code has been uploaded to GitHub.doi:<https://doi.org/10.5281/zenodo.14788957>.

Next is a screenshot of the operation results of the XGBoost model that didn't specifically handle ordinal response variables before.

From Table 2, we found that the prediction accuracy of Ordinal Forest model in R language [52] is better than that of the SVM, DT, and ANN models which do not consider the handling of ordinal response variable. However, its accuracy is equal to that of the XGBoost model which does not consider the handling of ordinal response variable.

So we added a function for handling ordinal response variable [49, 50] to the XGBoost model, the code has been uploaded to GitHub. doi:<https://doi.org/10.5281/zenodo.14788957>.

This time, we found the accuracy of the XGBoost model (add the handling of ordered response variable) was significantly improved. The following figure is a screenshot of the operation results of the XGBoost model with the handling of ordinal response variables added.

Since the XGBoost model (add the handling of ordered response variable) has demonstrated better prediction performance than other models, we still recommend the XGBoost model. All the subsequent figures in the text have been regenerated and modified according to the new XGBoost model.

Your suggestion has significantly improved the accuracy of our simulation and prediction. Thanks a lot!
