# Supplementary material for: What factors enhance students’ achievement? A machine learning and interpretable methods approach
Source: PLoS One. 2025 May 16;20(5):e0323345. doi: 10.1371/journal.pone.0323345 (PMC12083833; doi:10.1371/journal.pone.0323345)
Supplement: Attachment — Submitted filename: Response to Reviewers 3.27.docx [file pone.0323345.s005.docx]

Dear Editor and Reviewers,

Thank you for your valuable comments. Based on your feedback, we have made the necessary modifications to our manuscript. We hope these changes meet the publication standards of this journal.

Best regards,

Authors

**Editor Comments:**

1.In the abstract, clearly state the research gap in a sentence, include key results, and refine the conclusion to highlight major findings and implications.
AU comments: Thank you for your valuable suggestions! We have revised the abstract to prominently address four key aspects: the research gap, main methods, major findings, and implications (see lines 16–30).

2.The introduction should better define the study focus, objectives, and methodology for improved reader engagement.

AU comments: We appreciate this feedback. The introduction has been restructured to clearly outline the study’s focus, objectives, and methodology (lines 35–71).

3.Citation inconsistencies should be corrected by adhering to a single referencing style.
AU comments: hank you for noting this. We have standardized all citations to a single referencing style, with modifications highlighted in yellow in the Reference section.

4.better linkage in the literature review will improve coherence
AU comments: We greatly appreciate this suggestion. The literature review has been revised to strengthen logical connections between sections (see lines 134–135, 150–153, and 155–157).

5.minor grammatical errors
AU comments: Thank you for highlighting this. We have thoroughly proofread the manuscript to correct grammatical errors, with revisions marked in yellow.

**Reviewer #6:**

1. The manuscript contains some grammatical errors.

AU comments: Thank you for your observation. We have carefully reviewed the entire text and corrected grammatical issues, with changes highlighted in yellow.

2.Citation inconsistencies.

AU comments: We appreciate your attention to this detail. All references now adhere to a consistent style, as indicated in the yellow-highlighted modifications.

Insufficient linkage in the literature review.

AU comments: Thank you for this insightful suggestion. We have enhanced the flow and connectivity of the literature review (see lines 134–135, 150–153, and 155–157).
